# Transitioning financial responsibility for health programs from external donors to developing countries: Key issues and recommendations for policy and research

**DOI:** 10.7189/jogh.08.010301

**Published:** 2018-06

**Authors:** Stephen Resch, Robert Hecht

**Affiliations:** 1 Department of Health Policy and Management, Center for Health Decision Science, Harvard T.H. Chan School of Public Health, Harvard University, Boston, Massachusetts, USA; 2 Pharos Global Health, Boston, Massachusetts, USA; 3 Department of Epidemiology of Microbial Diseases, Yale School of Public Health, New Haven, Connecticut, USA; 4 Jackson Institute of Global Affairs, Yale University, New Haven, Connecticut, USA

In this paper we explain why the transition of financing responsibility for health programs from external donors to domestic governments is picking up momentum; highlight the main challenges that countries and donors face in achieving smooth transitions that preserve health gains; point to the key strategies and tools that should be used in assessing, preparing, designing, and monitoring financial transitions; and finish by outlining a recommended agenda for priority research in this area. We argue that the drivers of transition include health program maturity, economic growth in aid-receiving countries, and slowing growth in levels of international donor assistance for health. We identify several factors that make successful transition especially challenging, such as establishing expectations among all key parties about levels of funding that are reasonable and fair, aligning local and international priorities, mobilizing adequate and sustained domestic funding, and improving efficiency of service delivery. We discuss several important tools available to address these challenges and improve the planning and implementation of financial transition, including robust resource tracking, policy modeling and financial forecasting, and analysis of the sustainability of increased domestic financial commitments. We conclude by highlighting key recommended areas for additional research and stakeholder engagement and avenues to pursue in these areas.

## THE GROWING IMPORTANCE OF FINANCIAL TRANSITIONS IN DEVELOPING COUNTRIES

International support for health programs in the developing low- and middle-income countries has grown dramatically since the early 2000s, even though it has started to level off during the current decade. Philanthropic organizations such as the Bill and Melinda Gates Foundation (BMGF), bilateral programs like the US President’s Emergency Program for AIDS Relief (PEPFAR), and global health partnerships such as Gavi and the Global Fund to Fight AIDS, TB, and Malaria have become major players in the financing of disease control programs and health system strengthening. Since 2000, development assistance for health (DAH) has grown from about US$ 10 billion per year to over US$ 30 billion [1], reaching a cumulative total over US$ 350 billion. Over US$ 109 billion has gone to HIV programs [2], with US$ 24 billion invested in vaccination [3].

While these efforts have had a measure of success in achieving their stated mandates [4,5] their long-run sustainability and effectiveness are not guaranteed. The goal underlying most DAH is not to function as an emergency “band aid” or to operate as currently structured in perpetuity. Rather, it is to foster the widespread proliferation of effective scaled-up programs, integrated responsibly into functioning health systems which are owned, operated, and funded locally. Now, a decade and a half since the acceleration of DAH growth, we are seeing a first wave of programmatic transition with attention increasingly focused on shifting the financial burden of health programs from external donors to local stakeholders. While this trend can be viewed as consistent with long-run goals of development programs, it is not without risks.

## THE MAIN DRIVERS BEHIND COUNTRY TRANSITIONS

Broadly, the factors contributing to this momentum toward financial transition can be grouped into three categories:

**Figure:**
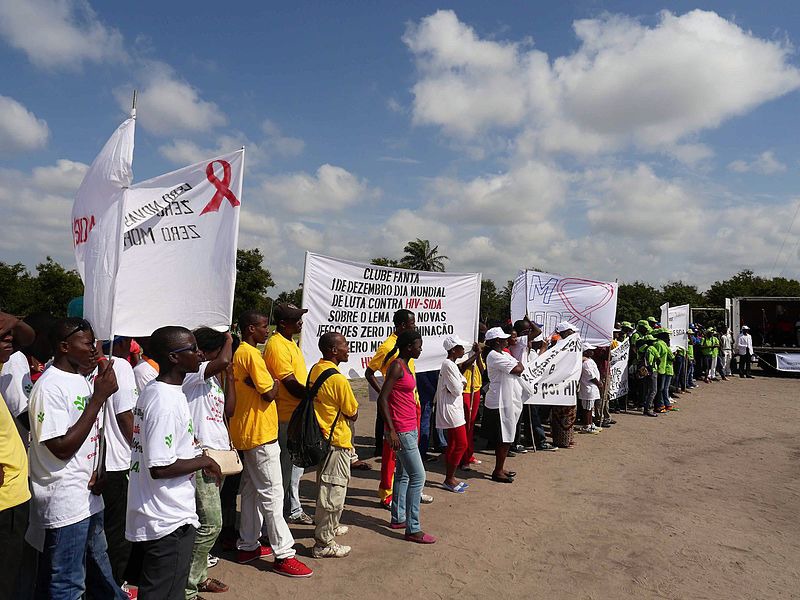
Photo: By Medici con l’Africa Cuamm (Beira, Mozambico, World AIDS Day 2013 auf flickr) [CC BY-SA 2.0 (https://creativecommons.org/licenses/by-sa/2.0)], via Wikimedia Commons.

First, some donor-supported health programs have met their initial goals for scaling-up and have matured into stable programs that may be ready for transitioning. In these circumstances, donors are increasingly eager to hand over these programs to reduce their long-term liabilities [6], as well as to concentrate aid in settings that most need it. BMGF’s Avahan HIV prevention program in India [7], Global Fund-backed AIDS treatment in Eastern Europe[8], Gavi’s support to introduction and widespread coverage of pentavalent, pneumococcal, and rotavirus vaccines, and USAID-assisted family planning programs in Latin America are some examples [9].

Second, most countries that have been recipients of health aid have experienced considerable economic growth and are losing eligibility for aid, or are perceived to be increasingly capable of financing health programs themselves [10]. For example, the 72 countries eligible for Gavi phase II from 2007 to 2010 achieved 50% higher GDP per capita by 2014. As a result, 21 of these countries have already transitioned or are moving toward it, with more to follow.

Third, levels of donor assistance for health alone are unlikely to increase at rates needed to meet ambitious new targets as encapsulated in the Sustainable Development Goals for 2030. The growth in health aid temporarily plateaued after the global economic crisis of 2008. Despite rebounding in recent years [1], the prospects for accelerated growth in aid levels are not encouraging. At the same time, health programs are stretching to achieve new and loftier targets. For example, despite unprecedented levels of health aid, less than 50% of all persons infected by HIV are on treatment, well short of the new 81% goal [4], and only a handful of Gavi-supported countries have introduced HPV vaccine nationwide to prevent cervical cancer, whereas WHO is recommending that most of these countries adopt the vaccine.

If program scale-up and health system strengthening is to continue, new resources must be found. While some advocates are focused on innovative approaches to increasing DAH such as financial transaction taxes or “world health insurance” [11], there has been a simultaneous effort to investigate whether DAH recipient countries can do more with domestic financing.

## KEY CHALLENGES FOR FINANCIAL TRANSITION

Based on our review of country experience [8,12,13] and our own work analyzing country transitions away from Gavi and PEPFAR, we see several key challenges countries and donors face in designing and implementing viable and sustainable financial transitions.

### Determining “fair share” for domestic financing

Determining countries’ “fair share” of program financing for HIV, malaria, immunization, and other health programs is not straightforward. Approaches include benchmarking against normative standards [14] such as the Abuja targets (15% of government expenditure allocated to health sector), as well as comparison to peer countries’ median effort [15] (eg, the UNAIDS Domestic Investment Priorities Index).

While these approaches provide useful guidance, many context-specific factors limit their utility for making policy prescriptions. Data on basic indicators such as government health spending are imperfect, and good estimates of domestic spending in particular disease areas are often not available. Even with accurate data, these indicators of “fair share” do not account for many possible mitigating factors beyond per capita GDP. Sound governance and solid institutions with the ability to collect revenue and reliably disburse government funds according to budgets do not always accompany economic expansion. Real capacity to finance health programs may thus lag economic growth.

### Aligning priorities

In many cases, analysis of financial needs for various high burden diseases suggests that the countries themselves could afford to pay more, taking into account their revenues – provided that they make health and key donor-backed programs as a priority. Many low- and middle-income countries are currently allocating less than 5% of GDP to health. Greater priority for the health sector in budget allocation process could enable many high-value health programs to expand considerably. Trade-offs are inevitable in budget allocations; decisions to shift government spending in favor of health will have to consider how to minimize the impact on other sectors. But in many settings, the magnitude of reallocation required for governments to replace donor financing and ensure continued program scale up does not appear to be overwhelming. In HIV, a recent analysis comparing expected GDP to expected resource needs for achieving UNAIDS’ Fast Track “90-90-90” goals found that in most countries the required funding amounted to less than 1% of GDP, even in high prevalence countries [16]. Resource shifts of this size are possible with strong political will. Moreover, because countries facing transition away from donor financings are those with a strong pattern of economic growth, some portion of the needed increase in government health spending can come from new fiscal space, rather than from reallocation away from existing projects in other sectors. Jamison et al. find that if even a modest share of projected economic growth in LMICs was allocated to health priorities, large health gains could be achieved without external funds [17].

The persistence of donor aid in countries that have the financial capacity to transition suggests that donor-supported health programs may reflect donor priorities more than country priorities, or that countries concentrate their own resources in priority areas where donor aid is less abundant [18]. Donors and country stakeholders need to align their priorities if transition is to occur without disruption to key health programs.

### Realizing efficiency gains

Plans for increasing reliance on domestic financing for health programs often optimistically anticipate efficiency gains. For example, Kenya's strategy for HIV financing assumes that the country will achieve efficiencies in the key cost drivers yielding a 30% reduction in resource requirements [19]. If these assumptions are to prove correct, donors and countries need to create an enabling environment. In some cases, there are opportunities for quick wins, particularly in the procurement of drugs and other commodities. However, most health programs are not managed in a way that prioritizes or rewards operational efficiency. Program managers typically do not have tools to measure or monitor their technical efficiency, and often do not have decision-making authority or incentives to innovate toward greater efficiency.

## STRATEGIES AND TOOLS FOR ENHANCING TRANSITIONS

Our review of good practices in transition risk analysis and planning [7,8,12,13] shows that countries facing transition away from external support of health programs, and donors seeking to exit without negative consequences can improve the probability of mutual success by following a collaborative and coordinated process for managing transitions. This process includes: pre-transition assessment of readiness; an agreed transition plan that proactively mitigates identified risks; a framework for monitoring the transition process; and a mechanism for ensuring accountability.

Transition plans should align with other simultaneous strategic changes in the health system (for example devolution of authority to lower level jurisdictions within the health system, or adoption of social insurance or other pooled health financing). Plans for transitioning financing should have incremental, verifiable milestones and mechanisms to foster accountability between external donors and national counterparts.

In the planning stage, a range of domestic stakeholders need to be identified and invited to participate. The Ministry of Finance, national budgeting agency, and legislative committees on health may be essential parties, in addition to Ministry of Health, when negotiating financial transition. Domestic advocacy and watchdog groups can be engaged too.

### Expenditure analysis, resource tracking, and costing studies

Expenditure analyses, such as those undertaken recently by PEPFAR country programs to complement the UNAIDS-sponsored National AIDS Spending Assessments [20] examining the flow of donor funds, are an essential input to planning financial transition. A similar analysis of domestic spending is also needed. Moreover, systems for routine resource tracking should be assessed and strengthened in preparation for transition, in order to ensure the implementation of financial transition plans can be monitored. Costing of key health services being transitioned [21] can also help to establish clear unit costs for future budgeting, and variations in costs across regions and individual facilities can also assist in diagnosing areas for future efficiency gains (see final section).

### Policy modeling and financial forecasts

Transition planning requires forecasts of expected program cost at least 5-10 years into the future, accounting for realistic and not purely aspirational programmatic goals. If project investments are likely to lead to a fall in disease incidence and prevalence, disease modeling should inform cost projections.

Forecasts of available resources under proposed financing schemes are needed. These projections must account for economic trends that may impact fiscal space. Proposals for innovative financing mechanisms such as trust funds, sin taxes, or other earmarked levies, should be accompanied by thorough analysis of their revenue generating potential, feasibility, and broader economic impact [22].

### Fair share analysis

**Figure 1:**
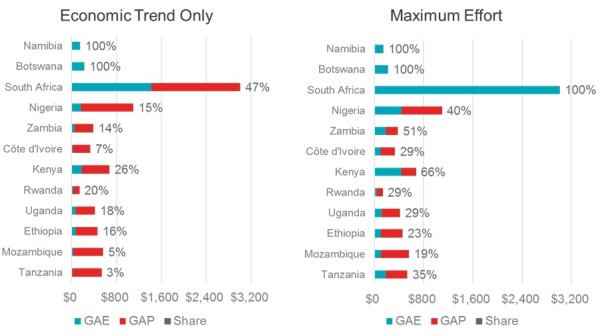
Analysis of the impact of increased domestic financing under “maximum effort” scenario in 12 PEPFAR (US President’s Emergency Program for AIDS Relief) countries indicates most will still have a financial gap to fill if they are to meet UNAIDS’ goals for program scale-up. Source: Adapted from [14].

As countries are growing, it is reasonable that some portion of additional GDP should go to health programs. Many countries are thought to be underspending on health generally, and some may not be distributing that spending optimally. In some settings, programs that have benefited from donor aid may be receiving proportionately less domestic resources. Establishing a plan for transition can benefit from examining a country’s current level and distribution of health spending in comparison to benchmark reference points such as the Abuja target or spending per PLHIV. **Figure 1** summarizes such analysis for the 12 original PEPFAR focus countries, and reveals that even if domestic financial effort for HIV was increased to a maximum level, there would still be a substantial gap in resources needed to achieve medium term goals for scale up in all but upper-middle income countries [14].

### Sustainability risks and incentives to mitigate them

The transition planning process should identify critical success factors and key risks. These might include vulnerability of program funding to political regime change, or the risk that integration with other health programs may result in reallocation of government spending towards other health priorities. A planning process should anticipate these risks and identify steps that can be taken to mitigate them. For example, legislation requiring a budget line for purchase of vaccines needed by the national immunization program (ie, “ring-fencing”) may be useful in some settings.

Donors and countries should negotiate the pace and scale of donor withdrawal. While countries have little direct leverage to stop the withdrawal of donor funds, donors are highly motivated to avoid letting programs falter. Even if the incremental steps are gradual, it is still important to establish a credible agreement with clear timelines and explicit milestones.

## AREAS FOR FURTHER ANALYSIS AND RESEARCH

To further strengthen tools and strategies for planning, managing, and evaluating global health transitions, countries and donors need to continue to invest in key areas of targeted data collection and analysis. Based on our review of the field, we argue that future policy research should focus on the following issues:

1. Fiscal space: It is important to improve the data and metrics for assessing country ability and willingness to pay for health services which were supported by donors prior to transition. Projected spending as a share of public sector health budgets is one measure, as are expenditure shares relative to disease burden. Cost-effectiveness analysis is also a useful tool for prioritizing programs, but work needs to be done to establish locally-derived valuation benchmarks of willingness-to-pay for improvements in health with which to evaluate alternative policy options, including health services previously funded by donors.
2. Efficiency: If financial transition plans rely on improving efficiency, it is critical to be able to search for these potential gains and use the appropriate data and tools. Projections of the likely savings from improved procurement and supply of key commodities (drugs, vaccines, diagnostics) and from shifting from costly health care workers to lower wage employees need to be conducted early in the transition and then tested against actual expenditures.
3. Aligning priorities and enhancing accountability: The process of managing imperfectly matched priorities between governments and exiting donors is difficult, as is enforcing performance that aligns with agreed transition plans and responsibilities. The recent use of scorecards and dashboards and of joint monitoring committees backed by solid monitoring data can help to stimulate a more positive dialogue between governments and donors and increase accountability. So can the involvement of civil society organizations as independent watchdogs equipped with data and analytical capacity[23].
4. Linking country transition with national health financing reforms: While transitioning away from donor support, many middle-income countries are simultaneously working toward health reforms including financing mechanisms to support Universal Health Coverage [24]. In this context, it is indispensable for country and donors to assess fiscal space, budgeting, and cost-effectiveness for transitioning services against the larger changes in health financing including guaranteed benefits packages, national health insurance, and provider payment reforms including performance-based financing.

To drive a strong analytical program on transitions, we propose that concerned donors such as the Global Fund, PEPFAR, and the World Bank should work together to define these key research topics, commission high quality analysis, and ensure that its main findings feed back into global and country practices. At present the transition policy analysis of these institutions is mainly fragmented and uncoordinated. This is an area ripe for expanded analysis, where investment by donors can have a large payoff in terms of more effective and sustainable transitions that preserve and extend health gains for billions of people living in low and middle-income countries, while contributing to more robust health systems.

## References

[1] Dieleman JL, Graves CM, Templin T, Johnson E, Baral R, Leach-Kemon K (2014). Global health development assistance remained steady in 2013 but did not align with recipients’ disease burden.. Health Aff (Millwood).

[2] Schneider MT, Birger M, Haakenstad A, Singh L, Hamavid H, Chapin A (2016). Tracking development assistance for HIV/AIDS: the international response to a global epidemic.. AIDS.

[3] Haakenstad A, Birger M, Singh L, Liu P, Lim S, Ng M (2016). Vaccine assistance to low- and middle-income countries increased to $3.6 billion in 2014.. Health Aff (Millwood).

[4] UNAIDS. Ending AIDS: Progress towards the 90-90-90 targets. New York; UNAIDS: 2017.

[5] GAVI Keeping children healthy: The vaccine alliance progress report 2015: Geneva.

[6] Vassall A, Remme M, Watts C, Hallett T, Siapka M, Vickerman P (2013). Financing essential HIV services: a new economic agenda.. PLoS Med.

[7] Sgaier SK, Ramakrishnan A, Dhingra N, Wadhwani A, Alexander A, Bennett S (2013). How the Avahan HIV prevention program transitioned from the Gates Foundation to the government of India.. Health Aff (Millwood).

[8] Gotsadze T, Fuenzalida-Puelma HL, Chkhatarshvili K, Chikovani I, Tabatadze M. Transition and sustainability of Global Fund supported programs: Sythesis report of selected country case studies and review. 2015. Curatio International Foundation.

[9] Cromer C, Pandit T, Robertson J, Niewjk A. The family planning graduation experience: lessons for the future. Washington, DC; LTG Associates Inc: 2004.

[10] Fan V, Savedoff B. The health financing transition: Inevitable change for the better? 2014. Available: http://www.cgdev.org/blog/health-financing-transition-inevitable-change-better. Accessed: 10 October 2017.

[11] Ooms G, Derderian K, Melody D (2006). Do we need a world health insurance to realise the right to health?. PLoS Med.

[12] Saxenian H, Hecht R, Kaddar M, Schmitt S, Ryckman T, Cornejo S (2015). Overcoming challenges to sustainable immunization financing: early experiences from GAVI graduating countries.. Health Policy Plan.

[13] Burrows D, Obeth G, Parsons D, McCallum L. Transitions from donor funding to domestic reliance for HIV responses: Recommendations for transitioning countries. 2016. APMG Health and Aidspan. Available: http://www.aidspan.org/publication/transitions-donor-funding-domestic-reliance-hiv-responses-%E2%80%93-recommendations. Accessed: 11 October 2017.

[14] Resch S, Ryckman T, Hecht R (2015). Funding AIDS programmes in the era of shared responsibility: an analysis of domestic spending in 12 low-income and middle-income countries.. Lancet Glob Health.

[15] Galárraga O, Wirtz VJ, Santa-Ana-Tellez Y, Korenromp EL (2013). Financing HIV programming: how much should low- and middle-income countries and their donors pay?. PLoS One.

[16] Piot P, Abdool Karim SS, Hecht R, Legido-Quigley H, Buse K, Stover J (2015). Defeating AIDS-advancing global health.. Lancet.

[17] Jamison DT, Summers LH, Alleyne G, Arrow KJ, Berkley S, Binagwaho A (2013). Global health 2035: a world converging within a generation.. Lancet.

[18] Hecht R. Shah R. Recent trends and innovations in development assistance for health. In: Disease control priorities in developing countries. Jamison DT, et al., editors. Washington DC: The International Bank for Reconstruction and Development / The World Bank: 2006. 21250298

[19] Kenya National AIDS Control Council. Kenya AIDS Strategic Framework 2014/2015 - 2018/2019.

[20] UNAIDS. NASA Publications and tools. Available: http://www.unaids.org/en/dataanalysis/datatools/nasapublicationsandtools. Accessed: 11 October 2017.

[21] Tagar E, Sundaram M, Condliffe K, Matatiyo B, Chimbwandira F, Chilima B (2014). Multi-country analysis of treatment costs for HIV/AIDS (MATCH): facility-level ART unit cost analysis in Ethiopia, Malawi, Rwanda, South Africa and Zambia.. PLoS One.

[22] Atun R, Silva S, Knaul FM (2017). Innovative financing instruments for global health 2002-15: a systematic analysis.. Lancet Glob Health.

[23] Rodríguez DC, Whiteside A, Bennett S (2017). Political commitment for vulnerable populations during donor transition.. Bull World Health Organ.

[24] Reich MR, Harris J, Ikegami N, Maeda A, Cashin C, Araujo EC (2016). Moving towards universal health coverage: lessons from 11 country studies.. Lancet.

